# 3D printed nerve guidance conduit for biologics‐free nerve regeneration and vascular integration

**DOI:** 10.1002/btm2.70057

**Published:** 2025-08-04

**Authors:** Jacob Schimelman, David B. Berry, Susie Johnson, Zhitian Ruskin Shi, Sophie Brown, Quyen T. Nguyen, Shaochen Chen

**Affiliations:** ^1^ Aiiso Yufeng Li Family Department of Chemical and Nano Engineering University of California San Diego La Jolla California USA; ^2^ Department of Orthopedic Surgery University of California San Diego La Jolla California USA; ^3^ Department of Otolaryngology‐Head and Neck Surgery University of California San Diego La Jolla California USA; ^4^ Shu Chien‐Gene Lay Department of Bioengineering University of California San Diego La Jolla California USA; ^5^ Department of Pharmacology University of California San Diego La Jolla California USA

**Keywords:** 3D printing, biologics‐free, biomaterials, hydrogel scaffold, nerve guidance conduit, peripheral nerve regeneration, vascularization

## Abstract

There is a clinical need for an effective nerve guidance conduit to treat peripheral nerve injuries. Many studies have explored different materials and active cues to guide neural regeneration, with some success. However, none have demonstrated a comparable or better functional recovery than the clinical standard autograft. Autografts are often insufficient for reconstruction of an injury to long nerves such as the sciatic or brachial plexus. Synthetic nerve guidance conduits (NGCs) have been investigated for these injuries to guide axonal regeneration and lead to functional recovery. We have designed a biologics‐free hydrogel‐based multi‐channel conduit with defined microscale features to guide axonal outgrowth. To investigate extraneural vascular infiltration and its effects on functional recovery, we also designed a multi‐microchannel conduit with defined regularly spaced micropores, orthogonal to the axon guidance channels. Using our custom‐built Rapid Projection, Image‐guided, Dynamic (RaPID) bioprinting system, we were able to fabricate each hydrogel conduit within minutes from a milliliter‐volume prepolymer vat. With our state‐of‐the‐art printing platform, we have achieved NGCs with a consistent channel wall width of 10 μm. We implanted the NGCs for 17 weeks in a murine sciatic nerve transection injury model. We assessed the functional recovery by dynamic gait analysis throughout the recovery period and by compound muscle action potential (CMAP) electrophysiology before NGC harvesting. Both the non‐porous and micro‐porous conduit groups led to functional nerve regeneration on par with the autograft group. Further, both conduit groups resulted in restoration of bulk motor function to pre‐injury performance.

**Translational Impact Statements**

We have developed a synthetic therapeutic that can be implanted to aid nerve regeneration after a debilitating injury that performs similarly to the current clinical standard treatment. One major hurdle to clinically translating an implantable therapeutic is needing to include biologics such as cells or active biomolecules, which increases the regulatory burden. With this in mind, we systematically developed a biologics‐free synthetic hydrogel nerve guidance conduit, composed of polymer backbones that have been used in many FDA‐approved applications, capable of restoring motor function.

## 1 INTRODUCTION

Peripheral nerve injuries (PNIs) can occur in everyday life, for example, from motor vehicle accidents; workplace accidents related to heavy machinery or power tools usage; and in combat‐related blast injuries such as from improvised explosive devices (IEDs). In the US alone, there are over 800,000 PNI‐related surgical operations performed annually.^1^ If left unrepaired, a PNI can result in chronic pain and/or paralysis. The current standard of care for reconstructive management of PNI in the clinical setting is a sensory nerve autograft.^2^, ^3^ Though autografts have been useful during reconstructive surgery, they are limited by donor site morbidity.^4^ Additionally, they are constrained by a potential mismatch between the length of the defect site and donor nerve. For major injuries to the brachial plexus or the sciatic nerves, the two longest sets of peripheral nerves in the body, using autografts may not be a viable approach.

Due to the above surgical considerations, there is intense interest in developing a synthetic nerve guidance conduit to replace the need for an autograft.^5^, ^6^ For over 20 years, there have been numerous studies published on investigating cylindrical polymeric conduits, with or without inner topological guidance, either by themselves or combined with a growth factor drug delivery treatment and/or cellular therapy.^5^, ^7^, ^8^, ^9^ Yet, none have resulted in an FDA‐approved system that has replaced the autograft as the standard of care for the surgical management of PNI. What has been approved are synthetic polymer hollow tubes or wraps to bridge the gap distance of the defect site, but they do not provide sufficient topological guidance or bioactive cues.^1^, ^10^, ^11^ There has been progress on commercializing decellularized human nerve allografts as an off‐the‐shelf alternative,^1^ but this will not fully meet the need of replacing autografts due to the inherent supply limitations of human cadaver donated nerves.

We have developed a compelling replacement to the autograft by systematically designing a nerve conduit to provide the minimum features necessary to lead to equivalent or better performance for motor function recovery in comparison to treatment by the reverse autograft. It has been well‐established that the microstructure provides topological guidance to the extending axon.^12^, ^13^, ^14^, ^15^, ^16^, ^17^, ^18^, ^19^, ^20^, ^21^, ^22^, ^23^, ^24^, ^25^, ^26^, ^27^, ^28^ Additionally, it has been shown that the stiffness and mechanical integrity of a conduit have a significant effect on the regeneration quality.^1^, ^29^

**FIGURE 1:**
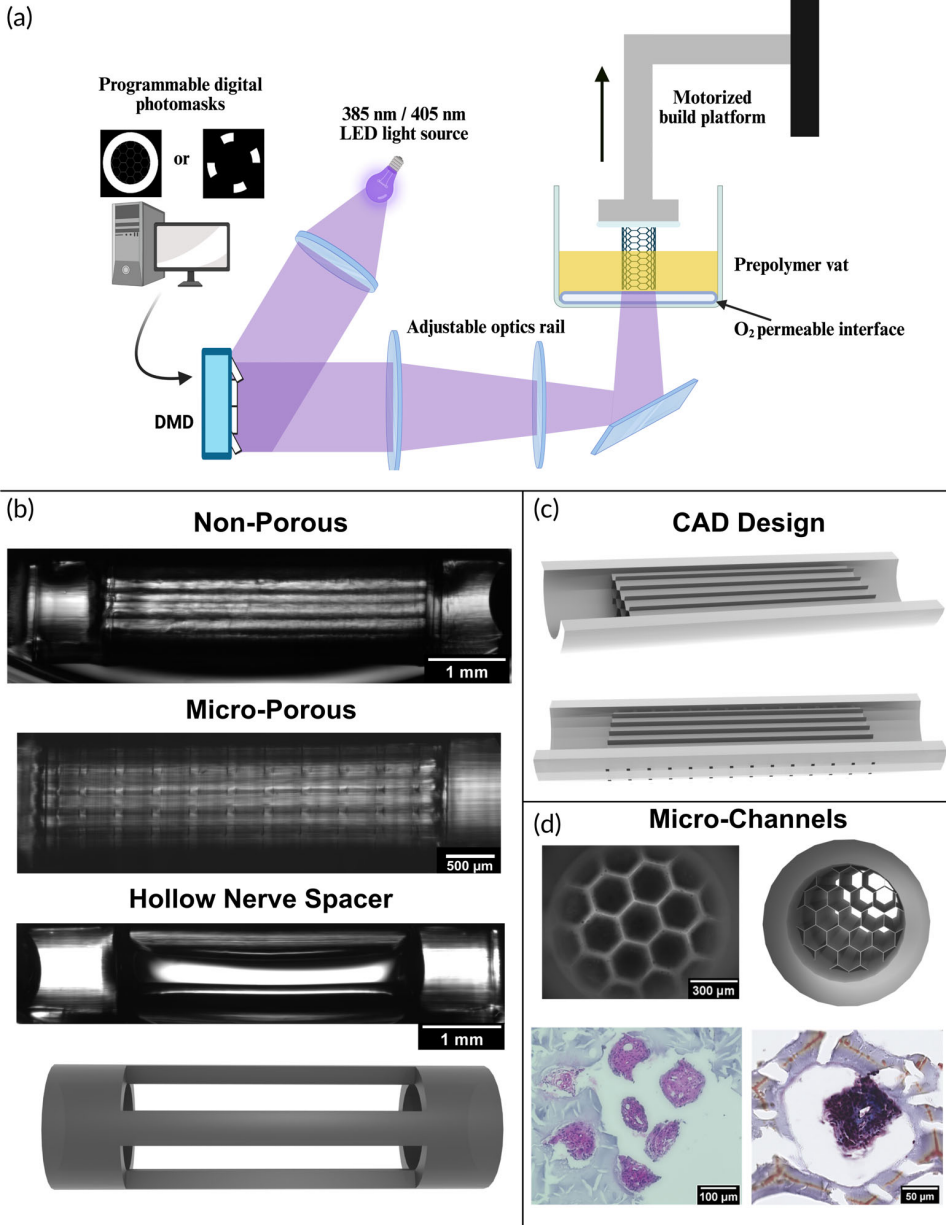
Development of a 3D‐printed microchannel nerve guidance conduit. (a) Schematic of the RaPID bioprinting system.^42^ (b) Longitudinal brightfield images of the non‐porous and micro‐porous NGCs and the hollow nerve spacer. Below the hollow nerve spacer image is its corresponding CAD rendering. (c) Longitudinal cross‐section renderings corresponding to the non‐porous (top) and micro‐porous (bottom) microchannel conduits. (d) Transverse cross‐section brightfield image (top left) and corresponding CAD rendering (top right) are shown. An H&E stained paraffin section (bottom left) showing representative fasciculated axon bundles extending through the seven central microchannels, and a Masson's Trichrome stained cryosection (right) showing a representative single fascicle extending through one of the central microchannels.

Our previous NGC design had four circular‐cross‐section microchannels that encouraged tissue regeneration across the length of the conduit of nerve bundles with multiple fascicles of axons.^29^ The conduits were produced via our custom Rapid Projection, Image‐guided, Dynamic (RaPID) 3D‐printing system (Figure 1a). We demonstrated that a photopolymerized composite hydrogel of poly(ethylene glycol) diacrylate (PEGDA) and gelatin methacryloyl (GelMA) was biocompatible within the murine sciatic nerve injury environment, with minimal to no evidence of an adverse inflammatory response. We optimized the conduit for mechanical integrity and stiffness, both for surgical handling and tissue stiffness matching, via tuning the concentrations of the prepolymer solution components as well as the printing parameters, such as the light intensity and print speed. This precise manipulation of mechanical properties without modifying the construct's geometric design is a unique manufacturing advantage of 3D printing. Additionally, there was preliminary evidence suggesting sensorimotor functional recovery by 6 weeks post injury (wpi). From this initial study, a 400‐μm‐diameter microchannel was shown to successfully guide axonal extension in vivo and the axons physically interacted only partially with the channel's inner surface area topography.

Most reports in the NGC literature have focused on axon guidance alone, but there has been recent interest in developing NGCs capable of enhancing intraneural blood vessel outgrowth within fascicles and/or extraneural blood vessel outgrowth from the distal tissue towards the injury site.^30^, ^31^, ^32^, ^33^, ^34^, ^35^ The extraneural vasculature is known to be activated by secreted VEGF‐A from the circulating macrophages that respond to the injury site, resulting in angiogenesis towards the regenerating axons.^36^ The blood vessels provide a topological bridge across the injury site for Schwann cell migration; thus, injury‐induced angiogenesis plays a critical role in innate peripheral nerve regeneration.^36^ However, although blood vessel outgrowth has been shown to be critical for nerve regeneration, it remains unclear how increasing the amount of blood vessels within a NGC affects tissue regeneration, and more critically, functional recovery.

One reason for the lack of translation of NGC research to the clinic is that they are generally fabricated using methods that are (i) difficult to scale, (ii) difficult to eliminate batch‐to‐batch variation, or (iii) not compatible with the high spatial resolution precision of microscale anisotropic features.^6^, ^37^, ^38^ Additive manufacturing, commonly referred to as 3D printing, provides a highly promising approach for producing polymeric biomaterials, for example, hydrogels, of high mechanical integrity and consistency at scale for off‐the‐shelf NGCs as well as for patient‐specific pre‐surgical planning designs and point‐of‐care manufacturing. The FDA has recently acknowledged the unique opportunity of implementing 3D printing solutions in the clinic and is currently working to standardize their assessments.^39^, ^40^ Another reason for the lack of clinical translation is that most NGCs reported include biologics such as neurotrophic or growth factors, guidance cues, and/or cellular therapies. Including bioactive materials, especially cells, within a biomaterial device greatly increases the regulatory hurdles (and cost) for commercialization.

In this report, we systematically study the development of NGCs capable of axon guidance, vascularization, functional nerve regeneration, and motor function recovery without the inclusion of any active drug or biologic to enable a path for direct clinical translation. Using our RaPID bioprinting system, we 3D print a PEGDA–GelMA composite hydrogel conduit with an array of 300‐μm‐wide microchannels separated by 10‐μm‐thin walls, where one conduit design version is functionally non‐porous and a second conduit design version has a patterned array of 50‐μm‐wide micropores that we hypothesize would enable extraneural vascular integration.

## 2 RESULTS

### 2.1 Nerve guidance conduit design

The conduit was designed for a complete transection sciatic nerve injury in a mouse model. This injury model resulted in the nerve ends retracting and forming a 4‐mm gap defect, as previously established.^29^ As such, the microchannel region of the conduit was designed to be 4‐mm long, and each end of the conduit featured a 1‐mm long hollow sleeve for securing each nerve end (Figure 1b,c). The microchannel diameter was optimized to be 300 μm to guide individual fascicles (Figure 1d). Additionally, to maximize the total number of microchannels for individual fascicle guidance, a tessellated hexagon pattern was chosen as the cross‐section geometry to increase packing density as compared to circular geometries, and the wall thickness between microchannels was minimized to only 10 μm (Figure 1d). To introduce micropores, we used computer‐aided design (CAD) software to add a regularly patterned array of micropores, spaced 250 μm apart in each direction, throughout the central conduit microchannel region (Figure 1b middle, c bottom). A square cross‐sectional geometry with a side length of 50 μm was used to ensure a sufficient entryway for nascent blood vessels.^41^ The RaPID 3D printing system readily produced this design change with precision (Figure 1b middle).

### 2.2 Development of a hollow nerve spacer negative control

To best evaluate the performance of the non‐porous and micro‐porous conduits, in addition to the positive control of the clinical standard autograft, a negative control is needed. A conventional option is to surgically perform the complete transection sciatic nerve injury without providing a therapeutic treatment. However, this approach suffers from the inability to control the positioning of the nerve ends during recovery. A simple hollow conduit would provide a direct control for topological guidance of the microchannels, but not a true negative control for the injury model. We solved this problem by designing and 3D‐printing a defined‐separation construct consisting of the same hollow sleeves from the conduit design that are joined together by four thin pillars, securing the nerve ends at the same 4‐mm gap distance as the therapeutic conduit groups (Figure 1b, bottom).

### 2.3 Mechanical testing

**FIGURE 2:**
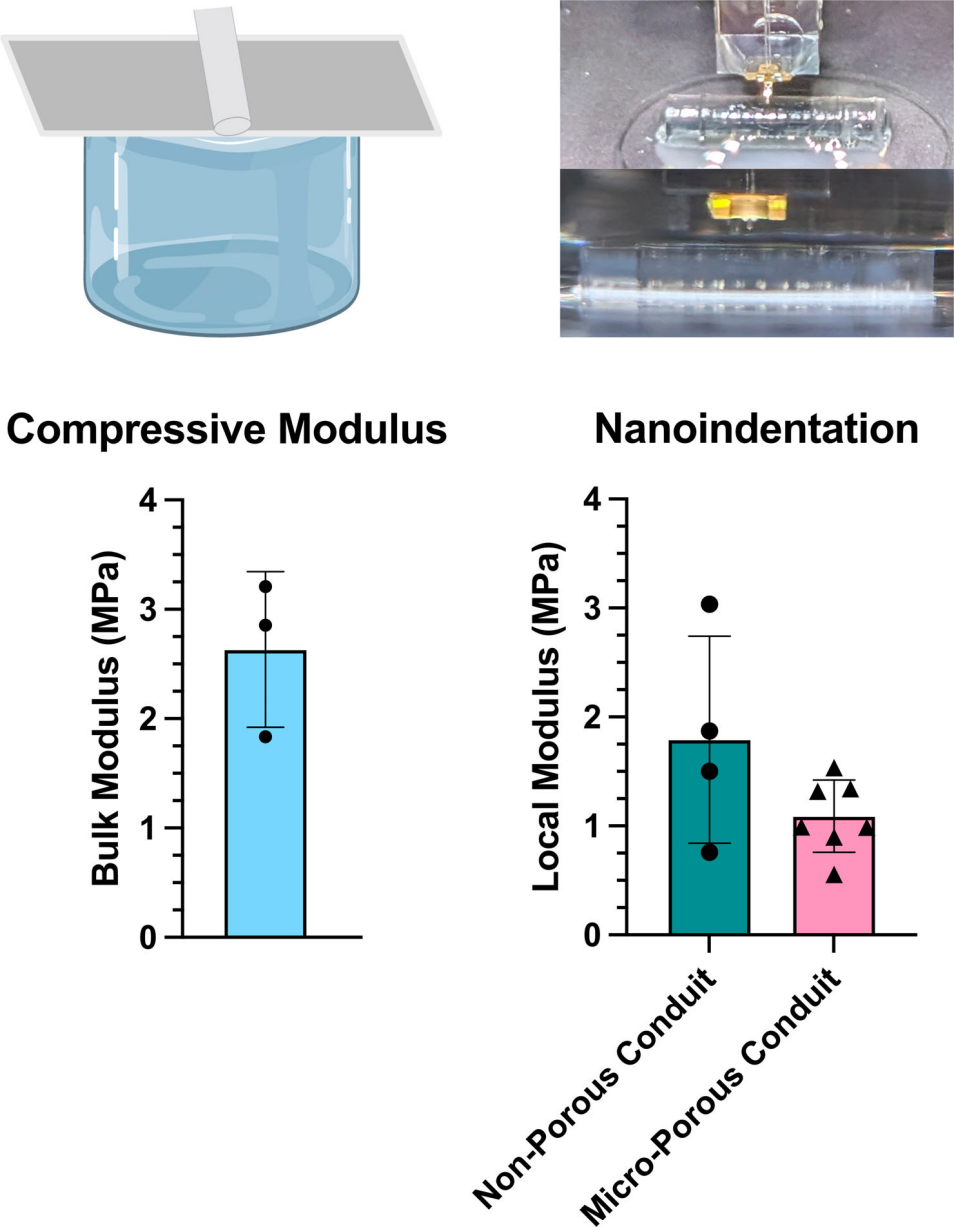
Mechanical testing. We performed both unconstrained compressive (left)^44^ and nanoindentation (right) testing to determine the bulk and local moduli, respectively. The nanoindentation was performed by sampling across the 4‐mm length of the central microchannel region of the conduit. All testing was performed in a room temperature 1X DPBS environment. The nanoindentation results found no statistical difference between the local moduli of the two conduit groups. Compressive testing: *n* = 3. Nanoindentation testing: *n* = 4 non‐porous conduit, *n* = 7 micro‐porous conduit. Each nanoindentation data point is an average of at least 10 independent measurements per specimen.

To ensure the Young's modulus of the conduit matches that of reported murine sciatic nerve tissue,^29^, ^43^ which is on the order of magnitude of 1 MPa, we performed unconfined compressive testing to obtain the bulk modulus of the 3D‐printed PEGDA–GelMA hydrogel material and nanoindentation testing to obtain the local modulus of each conduit design, with and without micropores, as printed (Figures 2 and S1). We found that the bulk modulus is 2.63 ± 0.71 MPa. The local moduli for the non‐porous and micro‐porous conduits were 1.79 ± 0.95 MPa and 1.09 ± 0.33 MPa, respectively, and were determined to be not statistically different.

### 2.4 Evaluation of non‐porous conduit

**FIGURE 3:**
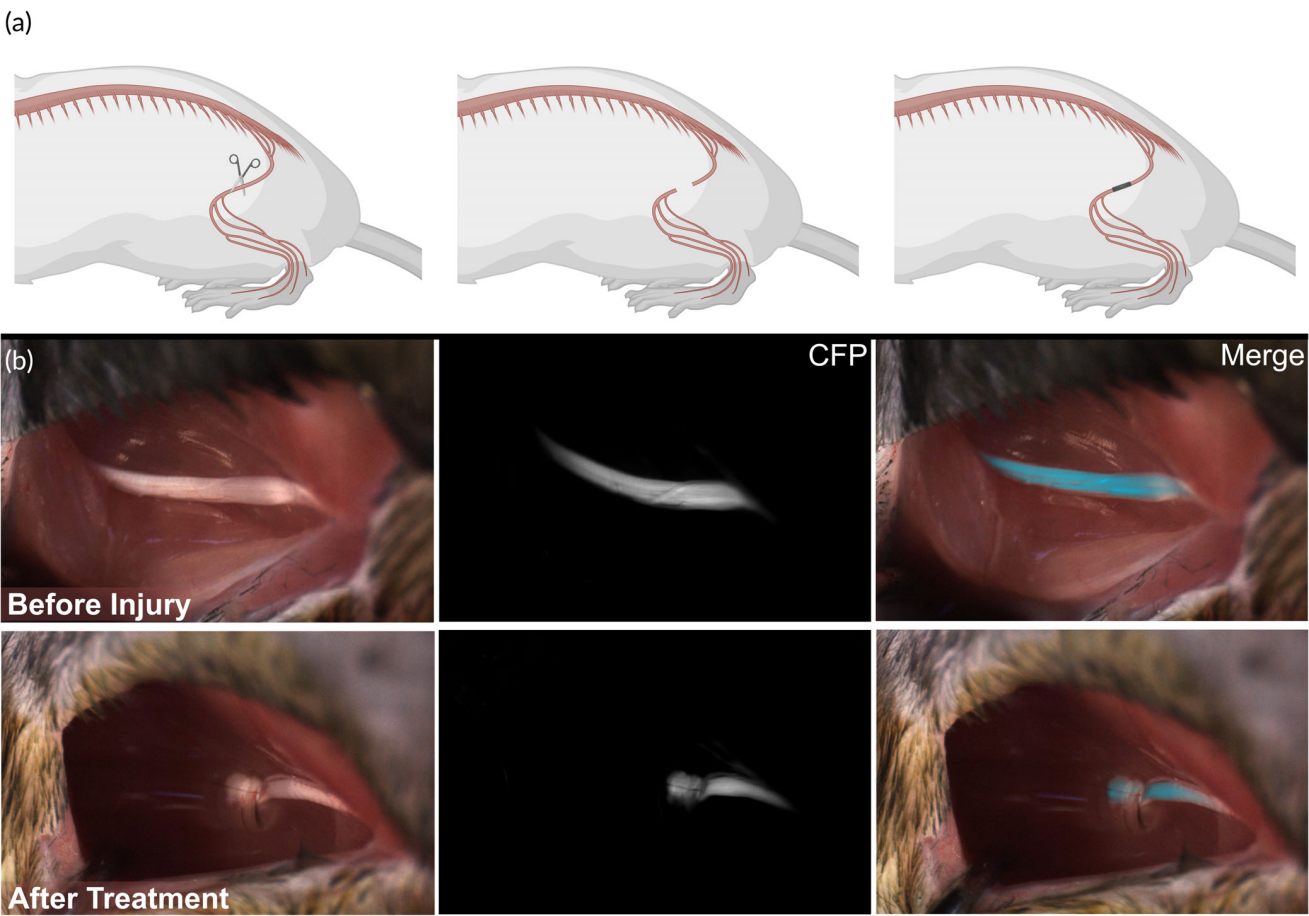
Complete transection sciatic nerve injury model and therapeutic implantation. (a) Schematic demonstrating the complete transection of the left hindlimb sciatic nerve via surgical scissors (left), retraction of the resulting two nerve stumps (middle), and implantation of conduit.^45^ The nerve stumps were secured within the conduit via a clinical‐grade fibrin adhesive. (b) White light, fluorescence, and merged images taken before and after complete transection of the sciatic nerve and implantation of the nerve guidance conduit.

**FIGURE 4:**
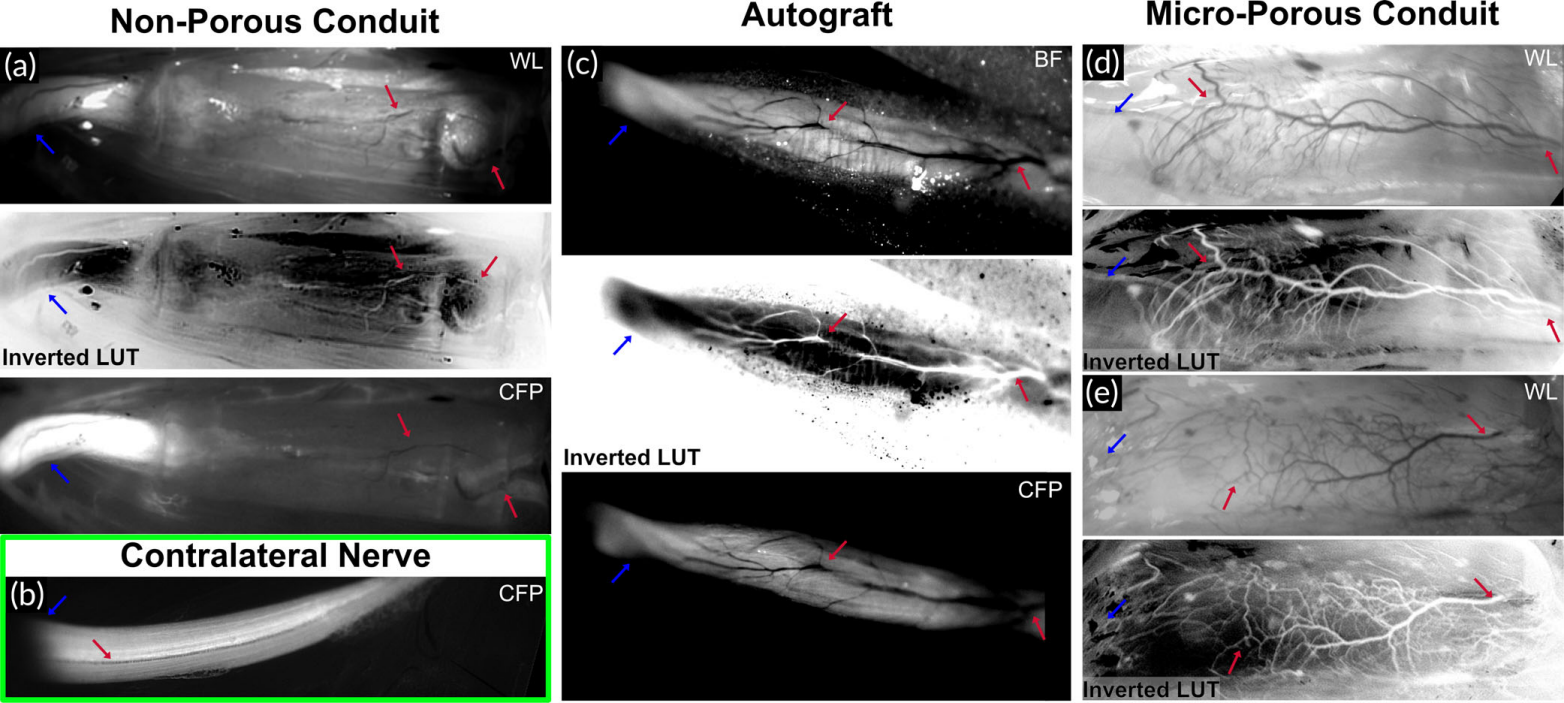
In vivo study of the therapeutic nerve guidance conduit. Two therapeutic conduit variants and a clinically employed therapeutic surgical intervention were assessed: non‐porous microchannel conduit (a), reverse autologous graft (c), and non‐porous microchannel conduit (d, e). White light, its corresponding inverted‐color processed image, and fluorescence in vivo images taken at 12 wpi (a, c). White light in vivo images and corresponding inverted‐color processed images taken at 4 wpi from two subjects (d, e). The inverted color images aid in differentiating the blood vessels from surrounding tissue and implant. Fluorescence in vivo image of a representative contralateral nerve at 17 wpi (b). Blue arrows: proximal end. Red arrows: distal extraneural vasculature growing out towards proximal regenerating axons.

A 12‐week in vivo pilot study of the hexagonal microchannel conduit design was used to compare its performance against the clinical standard treatment, a reverse autograft. The sciatic nerve was severed at a region prior to branching, and the conduit was immediately implanted and secured in place with a clinical‐grade fibrin adhesive (Figure 3). Thy1‐CFP transgenic mice enable the use of fluorescence in vivo microscopy to evaluate the axonal extension through the conduit microchannels in situ (Figure 3b). Multiple fasciculated axon tracts were fully extended from the proximal end to the distal end at 12 wpi. Notably, the axons extended through the central seven microchannels as individual fascicles (Figure 1d). We observed that the autograft resulted in dense external vascularization extending from the distal nerve tissue to the regenerating proximal axons (Figure 4c); this regenerated vasculature is morphologically distinct from the normal tissue physiology (Figure 4b). In contrast, little to no external blood vessels extended from the distal region along the conduit (Figure 4a). We hypothesized that the outer wall of the conduit shielded the regenerating axons from the pro‐regenerative external distal vasculature. We also hypothesized that introducing micropores along the conduit orthogonal to the microchannels would enable crosstalk between the injury site and the distal vasculature, thus facilitating the integration of the external distal vasculature with the conduit, and subsequently the regenerating axons.

### 2.5 Evaluation of micro‐porous conduit

A 4‐week in vivo pilot study was conducted to observe if the addition of the micropores in the size and pattern as stated led to a difference in extraneural vascularization. Indeed, distal vasculature extended along the length of the conduit and seemingly integrated with the regenerating axons (Figure 4d,e). The morphology of the extraneural vasculature interacting with the microporous conduit was highly similar to that of the autograft, indicating that the introduction of the micropores may lead to a more physiologically guided regeneration of the severed nerve (Figure 4c–e). Notably, the axon extension within the microchannels was similar to that of the non‐porous conduit, which alleviated any concern that the micropores could negatively affect the aligned guidance of the regenerating axons to the distal nerve end.

### 2.6 Four‐month study of functional recovery

The non‐porous and micro‐porous conduits with the positive and negative control groups were studied over a 17‐week recovery period to ensure sufficient healing time to assess functional recovery in a mouse sciatic nerve model.^46^ Dynamic gait analysis was performed longitudinally to assess the recovery of bulk motor function over time. Compound muscle action potential (CMAP) electrophysiology was performed at the end of the study. CMAP electrophysiology provides a direct assessment of the ability of the motor nerve tract to transmit an electrical signal to the muscle tissue.

**FIGURE 5:**
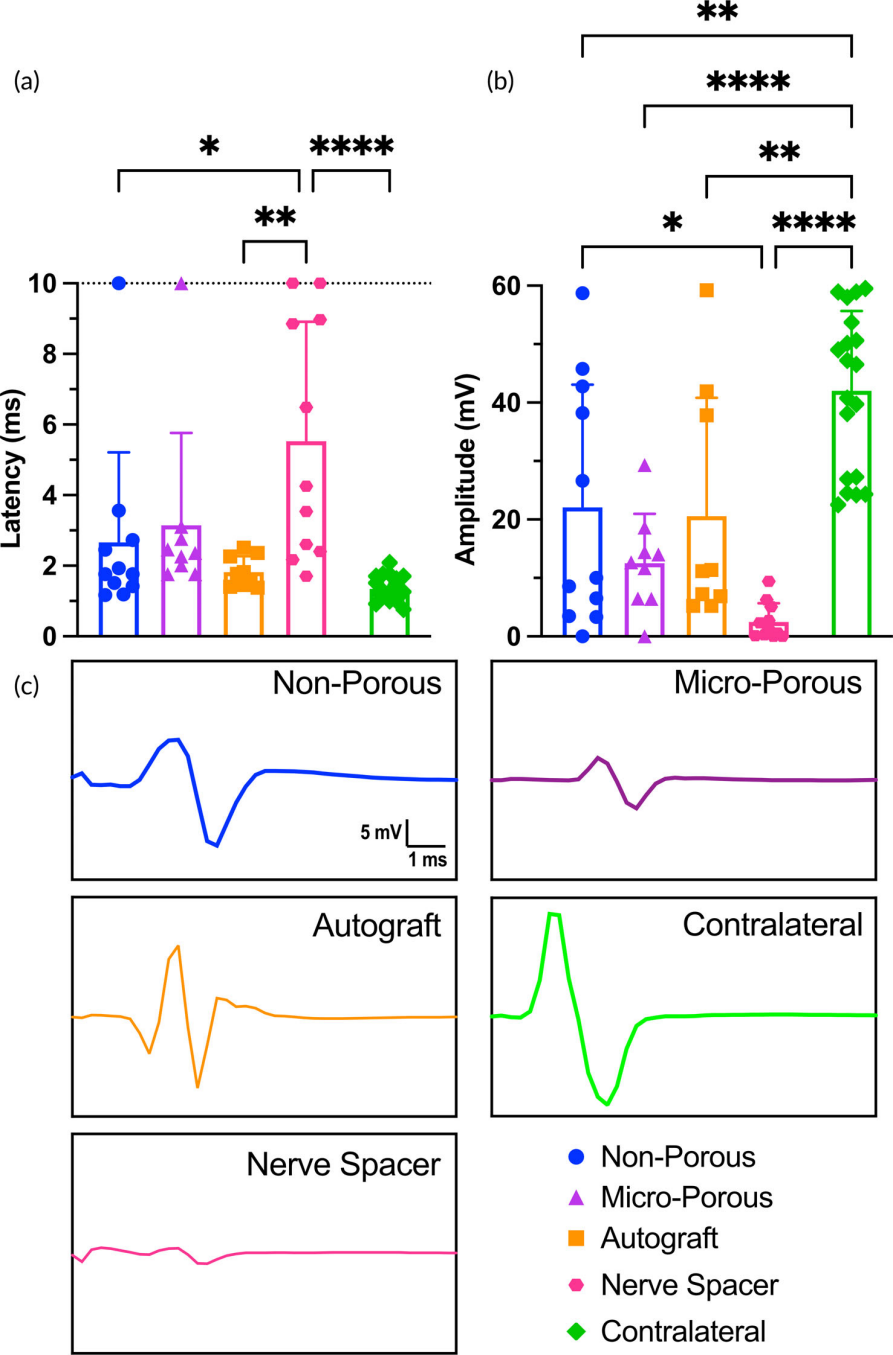
CMAP electrophysiology for assessing functional nerve regeneration. (a) Comparison of signal latency between the therapeutic conduit groups and control groups. A value of 10 ms was assigned to a waveform if no discernible signal was detected. (b) Comparison of peak‐to‐peak amplitude between the therapeutic conduit groups and control groups. A value of 0 mV was assigned to a waveform if no discernible signal was detected. Each data point is the average of at least three technical replicates; *n* ≥ 9. (c) Representative waveforms (i.e., closest to the mean for latency and/or amplitude) of each experimental group.

To perform the electrophysiology assessment, a dual prong electrode was inserted into the gastrocnemius muscle and a stimulator probe was put in direct contact with the nerve proximal to the injury. This test was performed with the sciatic nerve exposed to ensure that the proximal nerve region was directly stimulated. We characterized the latency and peak‐to‐peak amplitude of the response waveform to a 0.15 mA pulse stimulation (Figure 5). Latency is the time elapsed between the stimulation pulse and the start of the action potential signal (Figure 5a). Notably, both conduit treatment groups performed similarly to the autograft for both latency and peak‐to‐peak amplitude metrics; there were no significant differences between either conduit group and the autograft group (Figure 5a,b). The two conduit and autograft treatment groups all performed better than the hollow nerve spacer negative control. Representative waveforms that most closely matched the latency and peak‐to‐peak amplitude means from each group are provided (Figure 5c).

**FIGURE 6:**
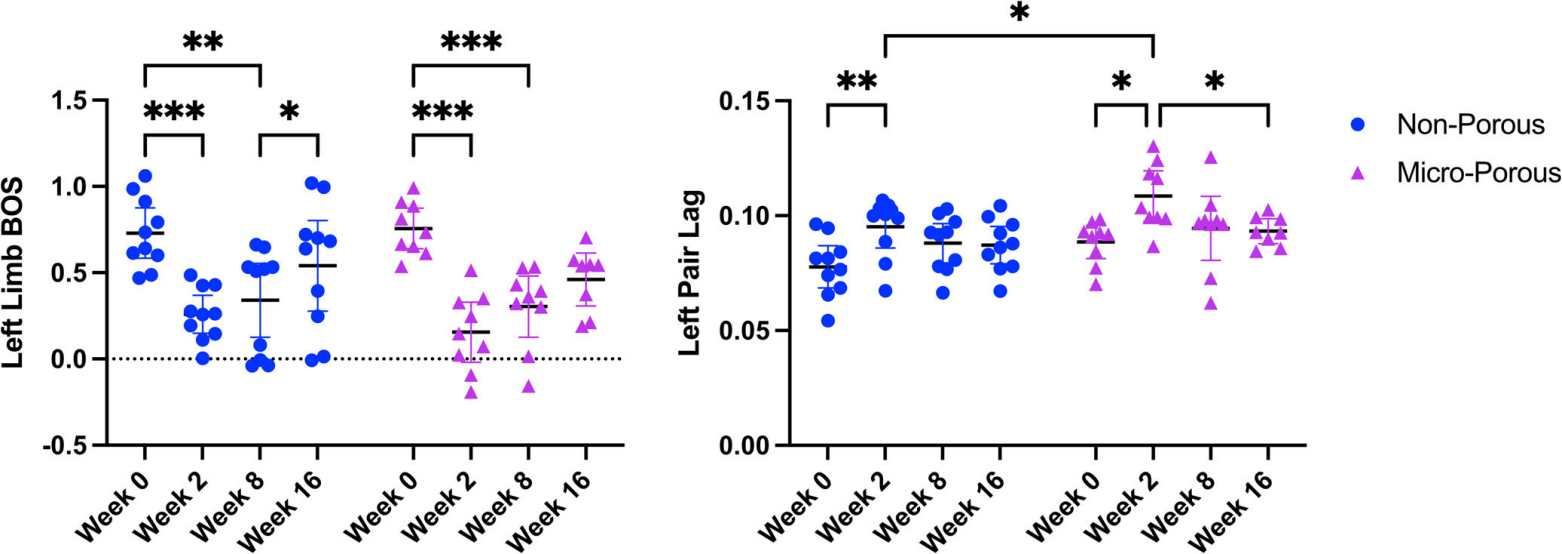
Dynamic gait analysis for assessing functional recovery after left‐hindlimb sciatic nerve injury. (Left) Comparison over time of the left limb base of support (BOS) metric for the two conduit groups. (Right) Comparison over time of the left pair lag metric for the two conduit groups. *p < 0.05; **p < 0.005; ***p < 0.0005. Week 0 testing was done immediately prior to the sciatic nerve injury and conduit implantation surgery.

We evaluated the recovery of normal gait as an assessment of motor function by characterizing two dynamic gait parameters: left limb base of support (BOS) and left pair lag (Figure 6). Base of support is a well‐established metric of stable gait,^47^ and time lags are a well‐established metric of quadrupedal limb coordination during dynamic gait.^48^, ^49^ As the injury affects the left hindlimb, we were interested in evaluating the left side. The non‐porous and micro‐porous conduit groups both experienced statistically significant changes in their gait performance in both metrics at 2 wpi. At 16 wpi, the non‐porous and micro‐porous conduit groups performance was both no longer statistically different than their normal gait performance prior to the injury, according to both the left limb BOS and left pair lag metrics.

## 3 DISCUSSION

There has been much interest in incorporating bioactive components such as growth factors and cells within conduits to bolster functional regeneration.^7^, ^50^, ^51^, ^52^ However, such an approach, even if efficacious, likely will be prohibitively expensive and will need to undergo a complex and rigorous FDA approval process as it would be considered a combination product. Ultimately, a NGC that incorporates biologics is unlikely to readily translate to the clinic to replace the need for autografts due to the cost and regulatory hurdles. Our team set out to develop a conduit whose regenerative guidance properties were solely derived from polymeric materials properties and topological cues, as such a device would have a streamlined pathway to FDA approval, and thus a high likelihood of successful clinical translation to provide therapeutic benefit to patients.

This study demonstrated that a 3D‐printed PEGDA–GelMA composite hydrogel conduit with 300‐μm‐wide hexagonal microchannels can guide the regeneration of multiple discrete axon fascicles across a complete transection sciatic nerve injury site in a mouse model (Figure 1) and facilitate recovery of nerve function as shown by CMAP electrophysiology (Figure 5) and recovery of motor function as shown by dynamic gait analysis (Figure 6). Demonstrating that an NGC can isolate multiple fascicles and thus could in principle guide them to different regions is a significant finding as this capability fulfills a critical unmet need in clinically treating complex injuries to highly branched nerves such as the facial nerves.

The RaPID 3D printing system enabled a systematic evaluation of the NGC engineering design. Upon the initial in vivo study with the non‐porous conduit and autograft therapeutic conditions, we discovered that, while the autograft experienced robust external vascularization from distal blood vessels (Figure 4c), the non‐porous conduit experienced minimal external distal blood vessel interaction (Figure 4a). Angiogenesis from distal blood vessels towards injured axons is a known pro‐regenerative response of the peripheral nervous system.^36^ We hypothesized that the observed difference between therapies in enabling distal angiogenesis from the extraneural blood vessels was caused by the open environment of the autograft versus the closed environment of the non‐porous conduit. To test this hypothesis, we modified the conduit design by adding 50‐μm‐wide micropores patterned along the central microchannel region of the conduit to produce the micro‐porous conduit (Figure 1b,c). The RaPID 3D printing system readily enabled this design change. This engineering workflow is highly conducive to point‐of‐care manufacturing for patient‐specific implants.

We observed that the micro‐porous conduit led to robust angiogenesis from distal external blood vessels towards the proximal regenerating axons, similar in density and morphology to the angiogenesis observed in the autograft group (Figure 4c–e). Interestingly, both the non‐porous and micro‐porous conduit groups resulted in similar functional nerve regeneration and full recovery of bulk motor function, as assessed by CMAP electrophysiology (Figure 5) and dynamic gait analysis (Figure 6), respectively. Notably, both NGC groups provided functional nerve regeneration that was not statistically different from that of the autograft group—the clinical standard for surgical nerve reconstruction—as demonstrated by the CMAP latency and peak‐to‐peak amplitude metrics (Figure 5). The similarity in functional recovery between the non‐porous and micro‐porous NGC groups may be due to the complete transection nerve gap length being 4 mm, which is a relatively short gap distance. The pro‐regenerative effect of the external distal blood vessel interaction may be more significant for longer‐gap injury models in larger animals and especially for clinical translation to humans. Thus, to better understand if there is a therapeutic improvement with the presence of micropores, a future study is needed with a larger nerve gap defect created by transecting out a multi‐millimeter segment of the nerve. This is likely to be prohibitive in a mouse model due to its size.^53^

We explored the effect of regularly patterned 50‐μm‐wide micropores on vascularization and functional recovery of the regenerating axons. Though the addition of the micropores resulted in the desired angiogenesis along the conduit and in the integration of blood vessels with the regenerating nerve fascicles, it did not result in an improved outcome in the functional recovery assessments when compared to the non‐porous conduit. This absence of enhanced functional recovery in the presence of external blood vessel integration has also been observed by another group in a rat sciatic nerve injury model with a 15‐mm defect, though their conduit was a hollow tube lacking micro‐topological guidance.^31^ Further studies of the microchannel conduit with varying micropore sizes, densities, and spatial patterns will be required to understand the role of angiogenesis from the distal external vasculature in sciatic nerve regeneration with respect to functional recovery in larger animal models.

## 4 CONCLUSIONS

Overall, this study has demonstrated that in a mouse complete transection sciatic nerve injury model, a 3D‐printed PEGDA–GelMA hydrogel nerve guidance conduit with precise topological features led to functional motor nerve regeneration. Providing precisely patterned micropores along the conduit surface enabled extraneural vascularization throughout the length of the conduit, which was morphologically similar to the vascularization observed along the autograft. Angiogenesis from the distal vasculature is known to play a critical role in innate peripheral nerve regeneration.^36^ Surprisingly, there was no statistical difference in functional recovery metrics between the non‐porous and micro‐porous conduit groups, indicating that for relatively small gap distances, the topological guidance from the NGC microchannels and the proximal nerve stump intraneural vascularization together are sufficient to sustain axonal regeneration. After a 4‐month recovery period, the therapeutic NGCs provided quantitatively similar performance in functional regeneration to that of the clinically used reverse autograft surgical treatment. Further, the NGCs resulted in restoration of bulk motor function to pre‐injury performance. This finding is remarkable in that the therapeutic conduit contained no bioactive or cellular components.

This 3D‐printed NGC was explicitly designed to maximize its potential for clinical translation. The technology behind the RaPID bioprinting system has been successfully commercially translated.^54^, ^55^ We are currently working towards obtaining investigational device exemption (IDE) approval from the FDA. In parallel, we also plan to apply for the FDA Early Feasibility Study (EFS) program, which would enable us to perform a small pilot study in human patients.

## 5 MATERIALS AND METHODS

### 5.1 Study design

This study aimed to investigate how microscale topology can effectively guide functional neural regeneration without the aid of biologics. We previously developed the RaPID 3D‐printing system to produce nerve guidance conduits with four 400‐μm‐diameter channels.^29^ In that study, we demonstrated the biocompatibility and axonal‐guidance efficacy of the PEGDA–GelMA hydrogel nerve conduit in a mouse sciatic nerve defect model. Based on this previous work, we hypothesized that reducing the microchannel diameter would maximize the contact area between the regenerating axon bundle and the microchannel. We also hypothesized that adding regularly patterned micropores along the length of the conduit would improve microvascular integration. The designed conduit of this study had 19 microchannels with hexagonal cross‐sections for increased channel density and inscribed in a 300‐μm‐diameter circle. To maximize the number of microchannels and reduce the spacing between regenerating nerve fascicles, the microchannel wall thickness was 10 μm. The nerve guidance conduit was specifically designed for a young‐adult mouse sciatic nerve complete transection injury model which results in a 4‐mm gap defect. The total length of the conduit was approximately 6 mm, accounting for the approximately 1‐mm‐long hollow sleeves on each end for securing the severed nerve endings via clinical‐grade fibrin glue. Two therapeutic conduits, the non‐porous and micro‐porous microchannel conduits, were compared against a reverse‐polarity 2‐mm‐long autograft and a 3D‐printed hollow nerve spacer (negative control). We used only male mice for this study to minimize the statistical variance as this was a first‐of‐its‐kind study comparing these novel conduit designs.

### 5.2 RaPID printing of conduits

Gelatin methacryloyl (GelMA), 85% degree of methacryloylation, was synthesized as previously reported from gelatin Type A, bloom 300 (Sigma‐Aldrich).^56^, ^57^ Poly(ethylene glycol) acrylate (PEGDA, *M*_*n*_ = 700 Da) was purchased from Millipore‐Sigma. Lithium phenyl(2,4,6‐trimethylbenzoyl)phosphinate (LAP) was purchased from TCI America. Tartrazine was purchased from Sigma‐Aldrich. The development of the rapid projection, image‐guided, direct‐printing (RaPID) 3D printing system was detailed in our prior work.^29^ All concentrations stated as percentages are %(*w/v*) unless explicitly stated otherwise. The prepolymer solution was composed of 25% PEGDA, 7.5% GelMA, 1% LAP, and 0.075% tartrazine in 1× Dulbecco's phosphate‐buffered saline (DPBS) solution. PEGDA provides physiological stiffness matching of native nerve tissue, GelMA provides integrin‐binding domains to facilitate cell attachment as it is derived from collagen, LAP serves as a cytocompatible water‐soluble photoinitiator, and tartrazine is a nontoxic water‐soluble photoabsorber.^58^ The photoabsorber tartrazine was included in the prepolymer solution to produce conduits with a 10‐μm channel wall thickness.

The conduits were designed using CAD software (AutoCAD, Autodesk, Inc.), exported as an STL file, and sliced into a set of digital 2D images via a custom MATLAB script. The dimensions for the conduit design variants are 6 mm in total length, 1 mm in sleeve length, 1.75 mm outer diameter, and 1.25 mm inner diameter. The treatment conduit variants have hexagonal microchannels defined by a long diagonal length of 300 μm and channel wall thickness of 10 μm. The microchannels are 4 mm long to match the gap distance that results from a complete transection injury of the mouse sciatic nerve. The micro‐porous conduit has a regularly patterned array of square micropores with a 50 μm side length spaced apart 250 μm longitudinally and circumferentially, orthogonal to the longitudinal microchannels. A negative control version of the conduit design was made by eliminating all but the two sleeve ends and maintained the same 4‐mm separation length via four equally spaced pillars, each having cross‐sectional dimensions of approximately 0.4 mm × 0.25 mm.

### 5.3 Mechanical testing

The bulk Young's modulus of the formulated hydrogel material was characterized via an unconfined uniaxial compressive test using a micro‐mechanical tester (MicroTester, CellScale Biomaterials Testing, Ontario, Canada). Cylindrical specimens were prepared with a diameter of 500 μm and a height of 500 μm. The strain rate was 2 μm/s. The Young's modulus was calculated from a linear regression analysis between 5% and 15% strain (*n* = 3).

The local Young's modulus of each microchannel conduit design was characterized via nanoindentation (Piuma, Optics11 Life, Amsterdam, Netherlands). For each specimen, a full‐length conduit was fabricated to the same specifications as the in vivo implants. The conduit was secured to a plastic Petri dish using a small dot of a commercial cyanoacrylate adhesive (Loctite 401), and the conduit was then fully immersed in 1× DPBS solution. The nanoindenter probe tip used was 58 μm in diameter, and the cantilever had a spring constant *k* of 4.580 N/m. A minimum of 10 (and a maximum of 30) independent measurements were made along each conduit. Non‐porous conduit: *n* = 4. Micro‐porous conduit: *n* = 7. The force–displacement curves were analyzed using the Hertzian model for hemispherical samples in the DataViewer software V2.6.0 (Optics 11 Life, Amsterdam, Netherlands). The Poisson's ratio was assumed to be 0.5.

### 5.4 Surgical procedures

We used male BL/6 mice purchased from Charles River Labs for the large 17‐week in vivo studies of the two therapeutic conduit designs (*n* = 10), the autograft (*n* = 8), and the nerve spacer negative control (*n* = 8). We used male transgenic Thy1‐CFP BL/6 mice (The Jackson Laboratories, USA) from inbred colonies for all other in vivo studies (including pilot studies of the two therapeutic conduit variants). All mice were between 15 and 18 weeks in age at the date of surgery. The transgenic mice expressed cyan fluorescence protein (CFP) in nerve tissue, allowing for in situ visualization of nerve regeneration. The mice were housed in a pathogen‐free vivarium maintained by the University of California San Diego Animal Care Program. All surgeries were conducted under isoflurane anesthesia (3% induction, 1%–3% maintenance). The conduits, autograft, and nerve spacer negative control were all secured in place with a clinical‐grade human fibrin sealant (VISTASEAL™, CAT# VST02, Johnson & Johnson Health Care Systems, Inc.). After surgical intervention, the incision site was sutured closed, and the analgesic buprenorphine was subcutaneously administered. No more than four mice were kept in the same cage.

### 5.5 Motor function analysis

Dynamic gait analysis was performed on the GaitLab (ViewPoint Life Sciences, Lyon, France). A single researcher performed all related mice handling, and they were blinded to the experimental groups during behavioral testing and data analysis. The mice were trained on the walking platform one to two times a day for 1–2 weeks before surgery. Training involved allowing the mice to familiarize themselves with the GaitLab platform and running across the walkway with a consistent gait at maximum speed multiple times per session. Healthy cohort data for analysis was collected at the end of training, prior to surgery. The two treatment conduit groups (*n* = 10) were tested once every 2 weeks, starting 2 weeks after surgery for 16 weeks.

### 5.6 Electrophysiology

Compound muscle action potential (CMAP) electrophysiology was performed 17 weeks postinjury, before the nerve specimens were harvested, in both the ipsilateral and contralateral sciatic nerves. Electrophysiology was performed using a NIM‐Response 2.0 nerve integrity monitor system (Medtronic Plc., Minnesota, USA). The recording electrode was inserted into the gastrocnemius muscle, and a ground electrode was inserted subcutaneously. The stimulating electrode was applied to the proximal nerve and delivered an electrical stimulus of 10–15 mA at 1 pulse/s with a 0.1 ms duration. A minimum of three replicate waveforms were obtained per nerve and averaged. The averaged waveforms were analyzed for latency and peak‐to‐peak amplitude based on prior reported protocols.^59^, ^60^

### 5.7 Statistical analysis

Statistical analysis was performed using GraphPad Prism (GraphPad Software, Inc., La Jolla, CA) and G*Power v3.1.9.6.^61^ An a priori power analysis test was performed based on preliminary mouse behavioral testing data (effect size *f* was 0.948) to determine the minimum group size needed to achieve a power of 0.8 and an α error probability of 0.05. The result of which was a group size of *n* = 5, which resulted in an actual power of 0.896. Descriptive statistics and their representation in figures were presented as mean ± standard deviation. Comparisons between two independent groups were conducted using Welch's unpaired *t*‐test. Outliers, when identified amongst repeated measurements, were removed using the ROUT method where *Q* = 1%. For comparing multiple groups, an ordinary one‐way ANOVA was performed. For comparing multiple groups over time, either a repeated measures two‐way ANOVA or mixed‐effects model with the Geisser–Greenhouse correction was performed. The two‐way ANOVA analysis cannot handle missing values, so a mixed‐effects model was used in its stead when missing values were present. All ad hoc multiple comparisons testing was performed using Šídák's with a 95% confidence interval.

## AUTHOR CONTRIBUTIONS

Jacob Schimelman, Quyen T. Nguyen, and Shaochen Chen conceptualized this study. Jacob Schimelman, Susie Johnson, Quyen T. Nguyen, and Shaochen Chen contributed to the methodology. Jacob Schimelman, Susie Johnson, Zhitian Ruskin Shi, Sophie Brown, and Quyen T. Nguyen contributed to the experimental investigation. Jacob Schimelman, David B. Berry, Zhitian Ruskin Shi, and Sophie Brown contributed to the formal analysis of the collected data. David B. Berry developed the Matlab script software to process the dynamic gait analysis data, and Jacob Schimelman contributed to the software scripting. Susie Johnson contributed project administration for the animal studies. Jacob Schimelman contributed writing the original manuscript draft and data visualization. Jacob Schimelman, David B. Berry, Zhitian Ruskin Shi, Quyen T. Nguyen, and Shaochen Chen contributed reviewing and editing the manuscript. Quyen T. Nguyen and Shaochen Chen contributed funding acquisition, resources, and supervision.

## FUNDING INFORMATION

This work was supported by the Eunice Kennedy Shriver National Institute of Child Health and Human Development (HD090662, HD112026) and the National Institute of Neurological Disorders (F31NS125986), both of the National Institutes of Health. This work was also supported in part by the National Science Foundation Graduate Research Fellowship (Grant No. DGE‐1650112).

## CONFLICT OF INTEREST STATEMENT

The authors declare no conflicts of interest.

## Supporting information


**FIGURE S1:** Representative mechanical testing loading‐unloading curves. (a) Representative stress–strain curve of the unconfined compressive test. (b) Representative load–displacement curves of the nanoindentation test for each therapeutic conduit design.

## Data Availability

The data and code that support the findings of this study are available from the corresponding author upon reasonable request.
