# Supplementary material for: 3D printed nerve guidance conduit for biologics‐free nerve regeneration and vascular integration
Source: Bioeng Transl Med. 2025 Aug 4;10(6):e70057. doi: 10.1002/btm2.70057 (PMC12617550; doi:10.1002/btm2.70057)
Supplement: Supplementary file 1 — FIGURE S1: Representative mechanical testing loading‐unloading curves. (a) Representative stress–strain curve of the unconfined compressive test. (b) Representative load–displacement curves of the nanoindentation test for each therapeutic conduit design. [file BTM2-10-e70057-s001.docx]

3D Printed Nerve Guidance Conduit for Biologics-Free Nerve Regeneration and Vascular Integration

Jacob Schimelman^1^, David B. Berry^1,2^, Susie Johnson^3^, Zhitian Ruskin Shi^1^, Sophie Brown^4^, Quyen T. Nguyen^3*^, Shaochen Chen^1,4*^

^1^ Aiiso Yufeng Li Family Department of Chemical and Nano Engineering, University of California San Diego; La Jolla, CA, 92093, USA

^2^ Department of Orthopedic Surgery, University of California San Diego; La Jolla, CA, 92093, USA

^3^ Departments of Otolaryngology-Head and Neck Surgery and Pharmacology, University of California San Diego; La Jolla, CA, 92093, USA

^4^ Shu Chien-Gene Lay Department of Bioengineering, University of California San Diego; La Jolla, CA, 92093, USA

* Corresponding author. Email: S. Chen (shc064@ucsd.edu), Q.T. Nguyen (q1nguyen@health.ucsd.edu).


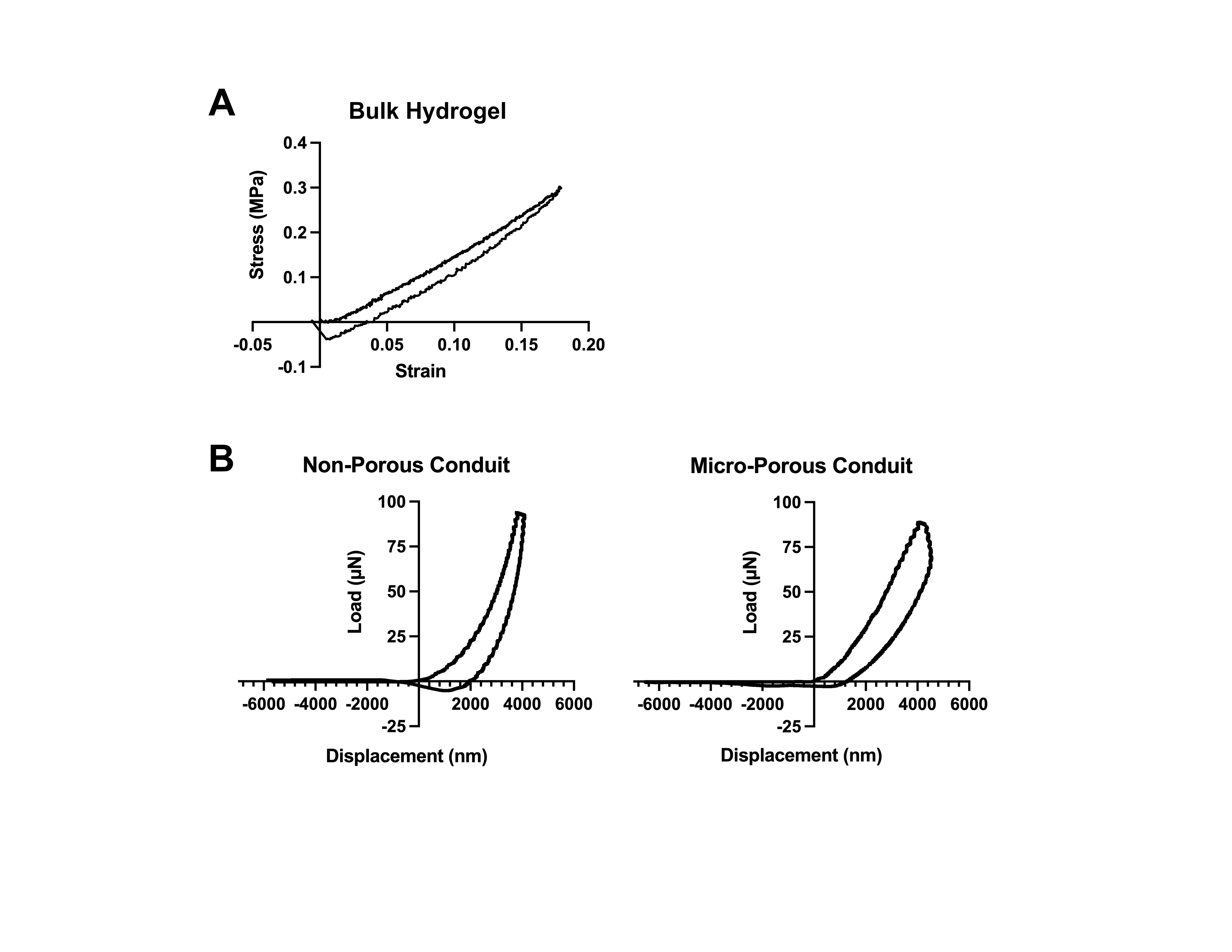


**Figure S1.** Representative mechanical testing loading-unloading curves. (A) Representative stress-strain curve of the unconfined compressive test. (B) Representative load-displacement curves of the nanoindentation test for each therapeutic conduit design.
